# Deciduous trees are a large and overlooked sink for snowmelt water in the boreal forest

**DOI:** 10.1038/srep29504

**Published:** 2016-07-12

**Authors:** Jessica M. Young-Robertson, W. Robert Bolton, Uma S. Bhatt, Jordi Cristóbal, Richard Thoman

**Affiliations:** 1 U.S. Geological Survey, Alaska Science Center, Anchorage, Alaska, USA; 2 International Arctic Research Center, University of Alaska, Fairbanks, Alaska, USA; 3 Department of Atmospheric Sciences, Geophysical Institute, University of Alaska, Fairbanks, Alaska, USA; 4 Geophysical Institute and Institute of Northern Engineering, University of Alaska, Fairbanks, Alaska, USA; 5 NOAA National Weather Service, Fairbanks, Alaska, USA

## Abstract

The terrestrial water cycle contains large uncertainties that impact our understanding of water budgets and climate dynamics. Water storage is a key uncertainty in the boreal water budget, with tree water storage often ignored. The goal of this study is to quantify tree water content during the snowmelt and growing season periods for Alaskan and western Canadian boreal forests. Deciduous trees reached saturation between snowmelt and leaf-out, taking up 21–25% of the available snowmelt water, while coniferous trees removed <1%. We found that deciduous trees removed 17.8–20.9 billion m^3^ of snowmelt water, which is equivalent to 8.7–10.2% of the Yukon River’s annual discharge. Deciduous trees transpired 2–12% (0.4–2.2 billion m^3^) of the absorbed snowmelt water immediately after leaf-out, increasing favorable conditions for atmospheric convection, and an additional 10–30% (2.0–5.2 billion m^3^) between leaf-out and mid-summer. By 2100, boreal deciduous tree area is expected to increase by 1–15%, potentially resulting in an additional 0.3–3 billion m^3^ of snowmelt water removed from the soil per year. This study is the first to show that deciduous tree water uptake of snowmelt water represents a large but overlooked aspect of the water balance in boreal watersheds.

Like the carbon cycle, the terrestrial water cycle contains large uncertainties that impact our understanding of local to global water budgets and climate dynamics. For example, increased global terrestrial water storage (~3200 billion m^2^ or gigatons, as reported by Reager *et al*.^1^) due to climate variability may have slowed a decade of sea level rise^1^. Yet, a limited understanding of the type, location, and climate-responsiveness of the different terrestrial water storage pools, including vegetation, contributes to large uncertainty in the “climate-driven land water storage” estimates (+/− 900 billion m^3^ of water)^1^. As described by Reager *et al*.^1^, understanding this uncertainty will require focusing on regions with significant freshwater export to the oceans and surface water hydrology that is rapidly and substantially impacted by climate change. Most of freshwater inflow into the Arctic Ocean is generated from the boreal forest^2^. Changes in storage – due to thawing permafrost, shifts in tree species and distributions, snow accumulation and ablation, and deepening of the seasonally thawed soil – has implications for streamflow and ultimately freshwater export from the boreal forest^2^. Of this list, the most under-examined aspect of the boreal water budget is tree water storage^2^,^3^. Further, a key uncertainty in the climate of boreal regions is the current and future state of net precipitation (precipitation minus evapotranspiration or ET)^4^. Transpiration composes a large fraction of ET^5^ and is not well constrained in boreal regions. Tree water use dynamics impact processes across multiple scales, from soil moisture and ecosystem water vapor efflux^6^ to regional climate^7^ and hydrology^6^,^8^. Many studies focus on transpiration but few on tree water content. However, water content is important for quantifying tree water storage, which impacts hydrology^8^,^9^, tree response to drought^10^,^11^,^12^, and the coupling of tree water use, soil moisture, and climate^7^,^8^,^12^.

The primary source for tree water storage, whether it is rainfall or snowmelt, has consequences for watershed water balance and the connections between tree water use, storage, and drought stress. Tree water storage can buffer the impact of drought on physiological activity. Existing studies found that trunk and stem water content sustains transpiration rates during dry (up to a week, in some cases) and wet periods alike^10^,^11^,^12^. A decoupling between transpiration and growing season rainfall may occur if snowmelt water is the primary water source for tree storage and utilization during the growing season. According to Yarie^3^, long-term experimental elimination of rainfall did not impact deciduous or coniferous upland tree growth in Interior Alaska’s boreal forest because of the implied reliance on snowmelt instead of summer rainfall. Aside from this, few studies have documented tree storage and utilization of snowmelt water.

Unlike transpiration, tree water storage is typically considered a minor aspect of the hydrological cycle and is therefore often ignored in hydrologic models^13^. Tree water storage is included in a vaguely defined error or storage term, which can include uncertainties associated with the other aspects of the water balance (precipitation, groundwater flow, evapotranspiration, and discharge). However, we hypothesize that tree water storage has the potential to greatly impact the water balance if the following three conditions are true. (1) The primary water source for trees is also a major component of the hydrological cycle, like snowmelt. In many mountainous semiarid regions, snowmelt is the dominant hydrological event of the year, resulting in greater streamflow, deep soil moisture recharge, and groundwater recharge compared to summertime rainfall^14^. Tree uptake and storage of snowmelt water may have a measurable impact on these hydrological processes. (2) The trees must have a high capacity to store and flux water in order to impact large-scale water balance. Trees with limited water storage capacity and/or low water use, such as some conifer species, will have a smaller impact compared to trees with high water use and storage, such as some deciduous species^8^. Finally, (3) trees cover a large enough part of the boreal landscape to have a measureable impact on soil water. Even trees with large water use and storage capacities would have limited impacts on watershed hydrology if they cover a relatively small area of land. Thus, hydrologic models representing watersheds or landscapes that contain tree species that can alter water balance may overestimate water export (via streamflow or groundwater flow/recharge) and underestimate ecosystem water flux and storage.

Greater attention must be paid to tree water storage dynamics in semiarid regions where water balance is snowmelt-dominated, and trees can potentially reduce water availability for streamflow and groundwater recharge. The boreal forest covers an extensive area of Alaska (~45% of the landscape) and western Canada, and much of the non-coastal areas have a semiarid climate (25–55 cm precipitation with 1/3^rd^ occurring as snow). Snowmelt is the most important aspect of the water balance because of the large volume of water that is released in a short period of time. For example, in and around Fairbanks, Alaska, historically ~30% of the annual precipitation is released over a 2–3 week period^15^. Much of the boreal forest is located in the zone of discontinuous permafrost, which impacts soil moisture pathways (including snowmelt-derived soil moisture) and the spatial distribution and types of trees present^16^,^17^. Areas with permafrost (generally along north-facing slopes and valley bottoms) typically have small statured coniferous trees (black spruce, *Picea mariana*), thick moss understory, thick organic soils (~20–25 cm) that are cold and wet, and a shallow seasonally thawed soil layer (~50–100 cm)^18^. Excess soil moisture that occurs during the snowmelt period moves laterally through the shallow subsurface soils, over the largely impermeable ice-rich soils and into streams^15^,^18^. Areas without permafrost (south-facing slopes) typically have deciduous trees (paper birch and trembling aspen, *Betula neoalaskana* and *Populus tremuloides*, respectively), often sparsely vegetated understory, relatively warm and dry soils with a thin organic layer (<10 cm), and fractured bedrock (typically deeper than 50 cm)^18^. It is assumed that *all* excess soil moisture, typically occurring during the spring snowmelt period, infiltrates the soil and eventually percolates to the groundwater^19^,^20^,^21^; the role of tree uptake and storage of the snowmelt water has not been considered as a pathway.

It is important to consider tree water uptake in both boreal systems (with and without permafrost) during snowmelt, when the trees are assumed to be either dormant or to not utilize enough water to impact soil moisture. The deciduous trees do not leaf-out until about 1–3 weeks after the conclusion of snowmelt. Similar to maple trees, birch trees are tapped during this pre-bud break period after snowmelt for their sap in order to produce syrup. Thus, boreal forest birch trees are clearly taking up and storing water prior to leaf-out. The assumption that this moisture is related to snowmelt water has not been rigorously explored. Further, little is known about black spruce water use dynamics during this period.

The goal of this study is to quantify tree water content during the snowmelt and growing seasons of two years with contrasting weather conditions. More specifically, we quantify the fraction of snowmelt taken up by trees at the plot level, and then scale our findings to the landscape-scale (Fig. 1). We also examine the implications of deciduous tree expansion for landscape-level tree water uptake. We conducted a field study on deciduous and coniferous trees in a watershed in Interior Alaska that is representative of many watersheds in non-coastal Alaska and western Canada. The field work was conducted at the Bonanza Creek Long Term Ecological Research area near Fairbanks, Alaska, from 2013 to 2014. Every week, we measured tree water content before and during the growing season in deciduous and coniferous-dominated ecosystems at two locations on a hillside (with different drainage properties) using Time Domain Reflectometry (TDR) probes installed in the trees^22^. We conducted all calculations within a Bayesian statistical framework in order to propagate uncertainty from the tree water content dataset to the scaling results.

## Results

The two years examined in this study represent an average rainfall year (~21 cm) with a delayed spring snowmelt and late leaf-out date (2013) and a high rainfall year (~48 cm) with average snowmelt and leaf-out dates (2014). Field measurements show that soil moisture reaches a maximum level near the end of the snowmelt period (5 cm depth ~32–34% in the deciduous ecosystem and ~58–60% in the coniferous ecosystem) (Fig. 2a,b). The higher soil volumetric water contents in the coniferous ecosystem are due to high organic matter content with greater water holding capacity and limited soil drainage^23^,^24^. The year with more rain (2014) showed higher soil moisture at 5 and 40 cm depths, particularly in the deciduous ecosystems (Fig. 2a). The temporal delay in increased soil moisture at 40 cm depth below ground surface in the coniferous/permafrost ecosystem is because the soils are frozen for a longer period compared to the 5 cm depths.

At the ecosystem scale associated with the research sites, both tree types (coniferous and deciduous) respond to snowmelt with increases in wood water content (Fig. 2c). However, the deciduous tree water content is over twice that of the coniferous trees (Fig. 2c). The high water contents of the deciduous trees have been observed in other studies focusing on birch^25^. The deciduous tree water contents also show greater seasonal variation compared to the coniferous trees (Fig. 2c).

### Tree uptake of snowmelt water

**Figure 1:**
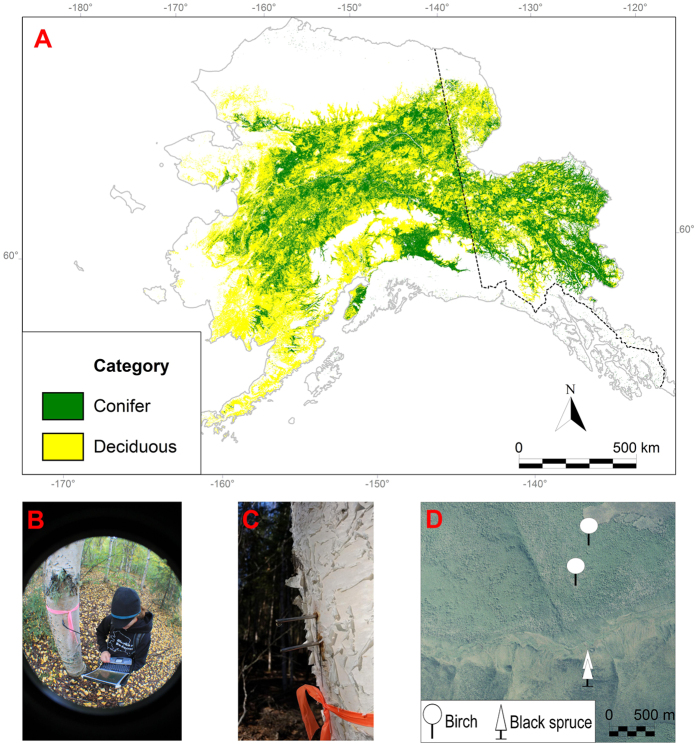
(**a**) A map of the area covered by deciduous and coniferous vegetation primarily in non-coastal parts of Alaska^24^. The red cross is the field study site at the Caribou Poker Creek Research Watershed (CPCRW). (**b**) J. Young measuring volumetric water content in a birch tree. (**c**) TDR probes in a birch tree. (**d**) Birch and black spruce field sites at CPCRW. This figure was generated using MiraMon v.7.1 (http://www.creaf.uab.es/miramon/Index_usa.htm).

The uptake of snowmelt water by trees is in reference to the snowmelt from within the ecosystems where the trees live. Our field data showed that the deciduous trees remove an average of 21–26% of snowmelt water and coniferous trees remove about 0.62–0.64% (Table 1). The landscape associated with the major boreal watersheds in Alaska and Western Canada has ~61.6% deciduous tree cover and ~38.4% coniferous tree cover (Fig. 1). We estimated that - over this land area - the trees take up an average of 17.8 to 20.9 × 10^9^ m^3^ of water at their maximum water content just prior to leaf-out (Table 1). This is ~ 98.3% more than the coniferous trees, which take up an average of 32.7 to 33.8 × 10^7^ m^3^ of water (Table 1).

### Post leaf-out transpiration of snowmelt water

Our field data showed that deciduous tree water content drops in the week after leaf-out (calculated on ~day 148 in 2013, ~day 121 in 2014) as the trees begin to transpire the stored water to the atmosphere (Fig. 3a). We applied the temporal trends in tree water content measured at our field site to the scaling calculations. Within the first week of the growing season with average rainfall (2013), 2.4–22% (12.4–2.2 × 10^9^ m^3^) of the stored water in the tree is transpired to the atmosphere (Table 2). From leaf-out to the 3^rd^, 5^th^, and 8^th^ weeks of the growing season, an average of 18%, 26%, and 29% of the stored tree water is transpired, respectively (water volumes presented in Table 2). Note that tree water content reaches the summer’s minimum (12.3 × 10^9^ m^3^ water or 72% of the maximum water content) in the 8^th^ week of the 2013 growing season (~day 205) (Fig. 3a). Within the first week of the high rainfall growing season (2014), 2.56% (3.2 × 10^8^ m^2^) of the stored tree water is transpired (Table 2). From leaf-out to the 2^nd^, 4^th^, and 5^th^ weeks, 5.15%, 8.33%, and 9.44% of the stored tree water is transpired, respectively (Table 2). Note that tree water content is at the minimum (18.9 × 10^9^ m^2^ water or 90% of the maximum water content) in the 5^th^ week of the 2014 growing season (~day 163) (Fig. 3a).

### Growing season patterns of tree water content

As observed from our field study, tree water contents show seasonal patterns, particularly in the deciduous-dominated ecosystems, with some variability between 2013 and 2014 (Fig. 3a,b). Our scaling calculations show that minimum water contents before snowmelt in deciduous dominated ecosystems are ~6.1 × 10^9^ m^3^ (2013) and ~6.4 × 10^9^ m^3^ (2014); in coniferous dominated ecosystems, the minimum water contents before snowmelt are 1.6 × 10^8^ m^3^ (2013) and 1.8 × 10^8^ m^3^ (2014) (Fig. 3a,b). During snowmelt, the deciduous tree water content increases as the trees become saturated (~100%, as a function of dry weight, observed in the field study) and our scaling calculations show that tree water contents reach ~1.8 × 10^10^ m^3^ in 2013 and 2.1 × 10^10^ m^3^ in 2014 (Fig. 3a). The coniferous trees also respond to snowmelt as water contents reach about ~50% (as a function of dry weight, observed in the field study), which is 3.4 × 10^8^ and 3.3 × 10^8^ m^3^ in 2013 and 2014, respectively (Fig. 3b). After leaf-out in the deciduous ecosystems, the average and minimum water contents are higher in 2014 given the record levels of rainfall received (~20.6 cm in 2013 versus ~48.2 cm in 2014) resulting in higher soil moisture. The minimum water contents during each summer are reported in the prior section. The scaled mean post-leaf-out water content of deciduous trees in Alaska in 2013 was 1.5 × 10^10^ and 2.0 × 10^10^ in 2014, which is 15.6% or 5.4 × 10^9^ m^3^ more water in 2014. Coniferous tree water contents remained relatively unchanged throughout the summer after snowmelt, with an average of 3.1 × 10^8^ and 3.2 × 10^8^ m^3^ in 2013 and 2014, respectively (Fig. 3b). This is 2.14% and 1.58% of the deciduous water content in 2013 and 2014, respectively.

## Discussion

In the boreal watersheds of Alaska and Western Canada, we estimated that deciduous tree uptake of snowmelt water prior to leaf-out represents a large (17.8 to 20.9 billion m^3^ of water), and previously unquantified, pathway of snowmelt water at the landscape scale. This amount of water is equivalent to 8.7–10.2% of the Yukon River’s average annual discharge (average annual flow (1976–2014) at the USGS Yukon River at Pilot Station). For perspective, recall that the global “climate-driven land water storage” estimate is 3200 (+/− 900) billion m^3^ of water^1^. Thus, the water uptake estimated by our study equates to 17.8–20.9 billion m^3^ of water, which is 0.55–0.65% of the global (3200 billion m^3^) or 1.9–2.3% of the uncertainty (900 billion m^3^) of the “climate-driven land water storage”.

It is assumed snowmelt water in deciduous tree dominated ecosystems, where permafrost is largely absent, mostly infiltrates the soil and percolates into groundwater storage^19^,^21^. Thus, it is also assumed that trees play a relatively insignificant role in the boreal hydrologic cycle, particularly during snowmelt. Permafrost-free areas in the boreal forest are where groundwater recharge primarily occurs because ice-rich permafrost acts as an aquitard, confining water to the surface soil layers^26^. Therefore, our study shows that deciduous trees, as a snowmelt water pathway and storage compartment, effectively remove up to 1/4^th^ of snowmelt water that was originally thought to recharge groundwater. Further, we show that coniferous trees remove and store little snowmelt water (Tables 1 and 2), which can generate extensive streamflow within these ecosystems^27^,^28^.

After leaf-out, the deciduous trees begin transpiring a large amount of snowmelt water, with potentially large impacts on the atmosphere. Prior to leaf-out, the atmosphere throughout much of Alaska and Western Canada tends to be relatively dry, but during and after leaf-out, atmospheric moisture significantly increases^29^. We estimated that the deciduous trees transpire 2–12% of the stored water during the first week after leaf-out, and 9–29% of the stored water between leaf-out and the period of minimum tree water content during the growing season (Table 2). Much of Alaska’s climate is semiarid with local sources of surface moisture representing a potentially large source of atmospheric moisture via evapotranspiration (evaporation and transpiration)^30^,^31^. Given that transpiration composes a significant fraction of evapotranspiration^5^, deciduous tree transpiration may play a substantial but unquantified role in the boreal forest’s summertime climate. At the time of green-up in Interior Alaska, there is still significant radiative heat loss during the night^32^; increased amounts atmospheric water vapor from tree transpiration is a greenhouse gas and can lead to warmer nighttime temperatures. Rigorous quantification of the role of boreal deciduous trees in the climate system is still needed given the potential for increased wildfire associated with increased transpiration activity. Increases in atmospheric moisture favor convective activity and cumulous cloud development that is associated with lightning and wildland fire, but not increased rainfall due to a cool mid-troposphere^2^,^4^.

**Figure 2:**
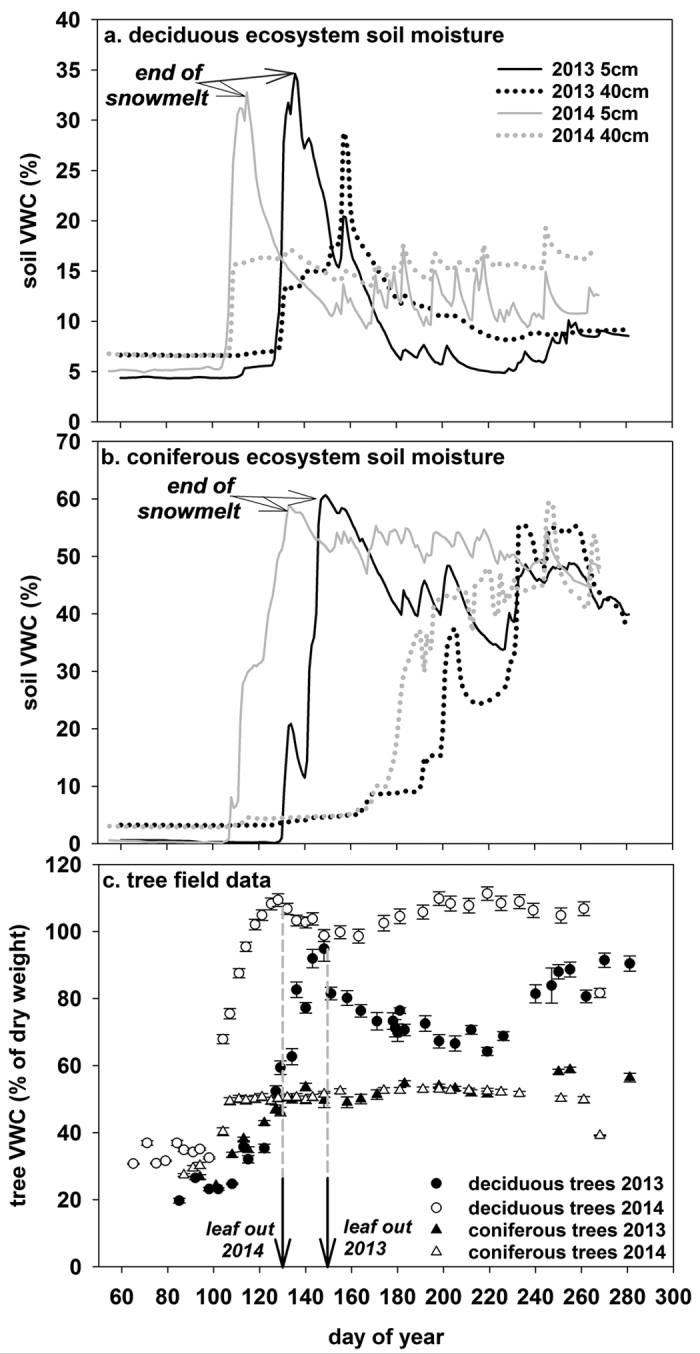
Field data collected from CPCRW research sites in 2013 and 2014. Soil moisture (volumetric water content, VWC, %) at 5 and 40 cm in the (**a**) deciduous ecosystems, (**b**) coniferous ecosystems, and (**c**) tree volumetric water content (VWC, % dry weight) measured on deciduous and coniferous trees. Arrows indicate the conclusion of snowmelt (when there is no snow remaining on the ground) and the approximate leaf-out days.

**Figure 3:**
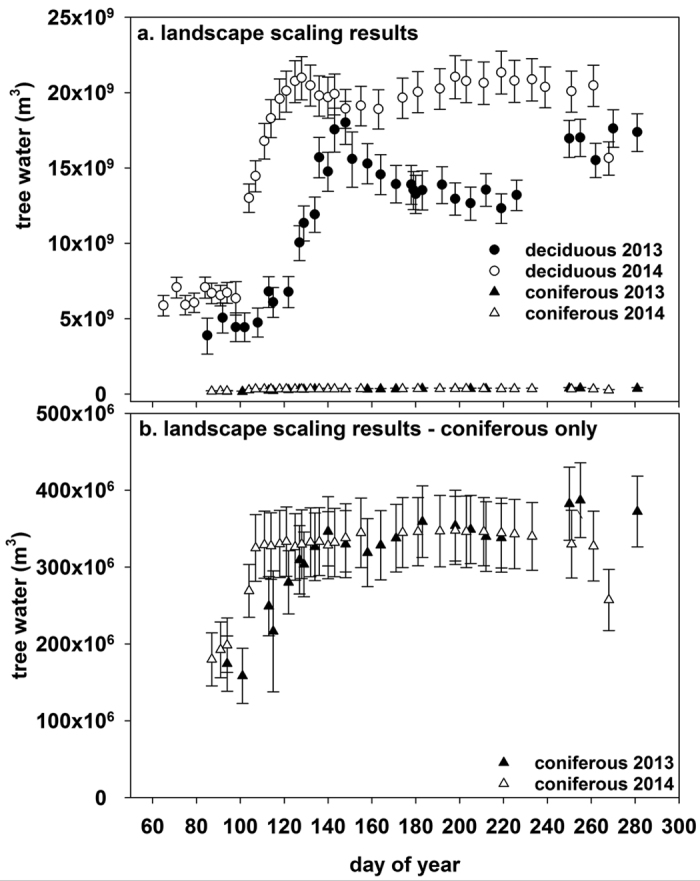
Mean and 95% credible intervals for tree water content scaled to the landscape level (m^3^ water) for 2013 and 2014 for (**a**) deciduous tree and coniferous tree dominated ecosystems and (**b**) only the coniferous ecosystem. Note the difference in scales between panels (**a,b**).

There are two primary differences in tree water content between the two years. In 2014, trees had higher average water content and water content reached saturation earlier compared to 2013 (Figs 2 and 3, Table 1). These differences are due to the record levels of rainfall received in 2014 (largest amount of rain received in 108 years of records), and the late conclusion of snowmelt (ending on day 140) and late leaf-out (day 150) in 2013. The average leaf out date near the study area ranges from day 131 to 138 (1974–2014), while the average last day of snowmelt in Fairbanks, AK, is day 118 (1997–2015 average from Remote Automated Weather Station data of the Alaska Fire Service). The high rainfall in 2014 resulted in a surplus of soil moisture, allowing the deciduous trees to remain at high water contents regardless of atmospheric demand. In 2013, however, soil moisture declined after the snowmelt peak and did not increase until significant rains occurred near the end of the growing season (Fig. 2). Thus, deciduous tree water storage declined while maintaining transpiration rates during periods of high atmospheric demand (personal observation). Differences between years, however, do not preclude drawing the general conclusions found within this study. This is because, despite differences, the trees in both years reach saturation levels in response to snowmelt (Fig. 2), take up a large fraction of snowmelt (Table 1), and maintain high wood water contents throughout the summer (Fig. 2).

Deciduous vegetation increases in its distribution in response to disturbance, such as wild fire. Wild fires are a natural occurrence in the boreal forest but the area burned and intensity of fires has been and will continue to increase with a warming climate^33^,^34^. It is estimated that fires may increase deciduous tree distributions 1–15% by 2100^34^,^35^,^36^. We estimated that over a range of potential increases in deciduous tree area -1%, 5%, 10%, and 15% - compared to current estimates of area covered by deciduous trees, 19.6, 20.4, 21.4, and 22.3 billion m^3^ of snowmelt water will be “diverted” from groundwater recharge in permafrost free areas, which is an additional 0.28, 1.05, 2.03, and 3.0 billion m^3^ of water, respectively (Table 1).

This study is the first to demonstrate how deciduous trees impact the water balance of northern ecosystems through diverting snowmelt water. This challenges hydrologists’ assumption that – in non-permafrost areas – snowmelt water predominantly recharges groundwater. We are also the first to show the potential consequences of wildland fire and the resulting vegetation succession for boreal water balance. Our findings suggest that in addition to directly drying the soil via thawing permafrost, fire may indirectly contribute to soil drying through promoting the establishment of deciduous vegetation. Lastly, this study is the first to quantify the volume of snowmelt water transpired by trees after leaf-out, with potential impacts on regional climate that may exacerbate wildland fire through increased convection and potential for higher lightning activity.

## Materials and Methods

We made five primary assumptions in these analyses. (1) Volumes of the trees at the research sites were calculated as cylinders. This assumption may result in some overestimation of tree volume because, near the top, there is some tapering of the trunk and branching. However, the branching is fairly significant and may compensate for the tapering. (2) The majority of the area determined to have deciduous tree cover is non-coastal Alaska, so we assume they are similar to our research watershed in Interior Alaska (Fig. 1). Thus, we assume that all the deciduous trees outside our research area attain the same level of wood saturation prior to leaf-out. Through personal observation, we found that the saturation phenomenon is easy to observe (via scraping off the bark or poking a hole in the tree and observing liquid water leak out), so widespread surveys of trees by simply puncturing the trees demonstrates that the “saturation phenomenon” is wide-spread. (3) The density of trees at our research sites is representative of stand densities in other parts of Alaska. We recognize that spatial heterogeneity in stand density exists, particularly due to topography. However, we are confident in our assumption because we conducted measurements on different topographic positions within the research watershed, capturing toe-slope and upslope stand densities. (4) In terms of the calculation and interpretation of water use between leaf out and the period of minimum water content, we assume the trees are primarily using snowmelt water. This assumption is supported by several lines of evidence. First, Yarie^3^ demonstrated that complete elimination of rainfall for 25 years did not impact deciduous or coniferous upland tree growth in Interior Alaska’s boreal forest because of the implied reliance on snowmelt instead of summer rainfall. Second, soil moisture measurements from 2011 to 2015 at our field sites show that rainfall events typically do not infiltrate to the 5 cm depths in the deciduous ecosystem soils. This is due to the 3–5 cm thick litter layer that inhibits moisture infiltration into the soil. The exception appears to be extremely wet years (2014). Third, the time period of interest (leaf out to minimum water content) had little to no rainfall in each year, so the available soil moisture was derived from snowmelt water. (5) We assume that the trees are taking up the current year’s snowmelt rather than stored soil moisture from the prior year. The soils in the deciduous ecosystems have low water contents prior to snowmelt^23^, as observed through coring (0–50 cm depths) and gravimetric water content measurements. Snowmelt in the current year is the only water source that is available in a large enough volume to completely fill the xylem of the trees.

### Field data collection

The research site is located at Caribou Poker Creeks Research Watershed (CPCRW, 104 km^2^ basin), ~48 km northeast of Fairbanks, Alaska. This site, established in 1969, is part of the Bonanza Creek Long Term Ecological Research (LTER) site (centered on 65°10′N, 147°30′W) and encompasses an area of 101.5 km^2^. CPCRW is located both within the boreal forest and the zone of discontinuous permafrost. Permafrost is generally found along north-facing slopes and valley bottoms^18^, and permafrost-free soils are typically found on south to southwest facing slopes. The thermal condition of the permafrost is unstable, varying from −3 to 0 °C, with thicknesses ranging from 0–120 cm^37^. The maximum active layer (seasonally thawed soil) thickness is ~52 cm at a low elevation point near the confluence of Poker and Caribou Creeks^15^. Black spruce (*Picea mariana*) is generally found along poorly drained north-facing slopes and valley bottoms. Aspen (*Populus tremuloides*), birch (*Betula papyrifera*), alder (*Alnus crispa*), and sporadic white spruce (*Picea glauca*) are found on the well-drained, south-facing slopes^18^. Tussock tundra (*Carex aquatilis*), feather moss (*Hylocomium spp.*), and sphagnum mosses (*Sphagnum sp.*) are also found along valley bottoms. CPCRW is part of the Yukon-Tanana Uplands that consist of metamorphic Precambrian mica-schists of the Birch Creek formation, mantled with Quarternary aeolian silts of varying thicknesses^38^,^39^. The soils are poorly developed silt loams, containing varying amounts of sand and gravel^18^,^40^. Overlying the silt loams are organic soils. In areas underlain with permafrost, low ground temperature reduces the rate of decomposition, resulting in an organic soil cover 20–50 cm thick^41^. In the warmer non-permafrost areas, the organic soils are no more than 15 cm thick^41^.

In March 2011, four sites were established in a mixed ecosystem watershed with areas dominated by coniferous trees on the north facing slope and areas dominated by deciduous trees on the south facing slope. On each slope, we established a site high and low on the hillside because of differences in drainage properties associated with topography (4 sites total) (Fig. 1d). During the winter at each site, we installed one soil pit with two soil moisture and two soil temperature sensors (Campbell Scientific, Logan, UT), one in the organic soil at 5 cm depth and one in the mineral soil at 40 cm depth. We also installed a meteorological station at each site, measuring relative humidity and air temperature, wind speed and wind direction, and precipitation. The deciduous trees created a relatively closed canopy (max LAI is 1–1.5 m^2^/m^2^), were ~17–20 m tall, and dominated by paper birch (*Betula neoalaskana*) with some trembling aspen (*Populus tremuloides*). The coniferous trees are exclusively black spruce (*Picea mariana*) 6–8 m tall and the canopy is open with a moss and shrub understory.

Tree TDR (Time Domain Reflectometry) measurements were modeled after Wullschleger *et al*.^22^. The measurements were taken on 40 deciduous and 30 coniferous trees in the 2013 and 2014 growing seasons, with 20 deciduous trees at each of the upper and lower south facing slopes and 15 coniferous trees on the lower and upper north facing slopes (Fig. 1d). The TDR data were averaged for the trees at the upper and lower sites on each slope (and, therefore, tree type) for the scaling calculations. Using a drill and a guide, stainless steel (non-magnetic) welding rods, 10 cm long and 3.18 mm in diameter, were installed with a hammer to 8 cm depth in the deciduous trees and 2.5 cm in the coniferous trees, about 0.5 to 1.5 m high. The difference in the depth of the probes was because the coniferous trees are small (~25 cm diameter at breast height or DBH) compared to the deciduous trees (~56 cm DBH). Every 1–2 weeks starting in March prior to deciduous tree leaf-out, TDR measurements (TDR100, Campbell Scientific, Logan, UT) were conducted on the trees at roughly the same time each day. The TDR was interfaced with the probes in the trees using a 1 m long 50 Ω coaxial cable with a male BNC fitting on one end (which connects to the TDR100) and stereo headphone connectors soldered to the other end of the cable (which was stripped and split into the center copper wire and the external wires). We extracted the peak and trough from the TDR waveform and calculated the apparent probe length and apparent dielectric constant (Ka value). For each observation, we converted the Ka value to volumetric water content based on the empirical equation developed via lab calibration. We conducted two rounds of lab calibrations, wherein we cut down a tree of each species, and cut each tree into five equal length segments. The welding rods were installed into each segment at the same depths in the wood as the trees in the field. As the tree pieces naturally dried down at room temperature over several months, TDR wavelengths and weights of each segment (g) were measured. When the segments were dry (and the weights ceased to change), the volume of each segment was measured by volume displacement in a tub of water. The gravimetric water contents were converted to volumetric water contents, and then plotted against the Ka values determined from the TDR wavelengths.

### Calculations

For each calculation, uncertainty was propagated from the field data (tree TDR, circumference, tree volume, SWE) to the final estimates of water volumes at the different scales by utilizing a Bayesian statistical approach and integrating all the datasets (utilizing the OpenBUGS software). For example, the uncertainties associated with field observations of tree volumes and maximum tree water contents are propagated into the calculations for tree water volume prior to leaf-out. The estimated volumes are then scaled with the area of each ecosystem type at the landscape-level.

We determined the relationship between tree volume (V) and circumference (C) for the trees containing the TDR probes of at our research sites, wherein for each ecosystem type (*eco*): *V* = *C* * *a*_eco_ + *b*_eco_. The parameters *a* and *b* were estimated (mean and 95% credible interval, *a*_*deciduous*_ = 2.39 [2.19, 2.65], *a*_*coniferous*_ = 2.81 [2.57, 3.04], *b*_*deciduous*_ = 3.38 [2.38, 4.23], *b*_*coniferous*_ = 1.49 [0.77, 2.25]) and utilized to determine tree volume/m^2^ ground area in the entire research site for which we measured circumferences of the remaining trees (900 m^2^ in the deciduous ecosystems and 400 m^2^ in the coniferous ecosystems). The tree volumes were summed for each ecosystem type to get the total volume of trees and then divided by the ground area to get tree volume/ground area (m^3^/km^2^ for most of the following calculations). We determined the maximum wood water content (m^3^ water) for each ecosystem type by: (1) averaging the maximum water contents for the 2–3 measurement days prior to leaf-out for each ecosystem type (deciduous and coniferous) in each year, (2) multiplying these data by tree volume (m^3^/km^2^), and (3) multiplying these data by the area of covered by deciduous and conifer ecosystem types in Alaska and the portion of the Yukon River watershed shared with Western Canada^42^. These vegetation types were obtained by a category reclassification of the Land Cover v.04 developed by Scenarios Network for Alaska and Arctic Planning (2005)^24^ using map algebra. To determine the volume of water transpired by the trees after leaf-out, we subtracted the water content for a given measurement day (shown in Table 2) from the maximum water content prior to leaf-out. For example, if time (*t*) 1 is the period of maximum water content and *t* = 3 is the measurement day within the first week after leaf out, then the volume of water transpired to the atmosphere is WC_t=1_–WC_t=3_. Table 2 shows the measurement days represented in the calculations for water volume transpired to the atmosphere. We calculated the percent of snowmelt water taken up by the two tree types prior to leaf-out as:

*% snowmelt*_eco,year_ = (1 − ((*SWE*−*tree.water*_eco,year_)/*SWE*))*100, wherein % snowmelt is calculated for each tree or ecosystem type (*eco*; deciduous, coniferous) and *year* (2013, 2014); *tree.water* is the estimated maximum tree water content (cm) from the field data. *SWE* is the estimated mean end of season SWE data averaged for the 1971–2000 period for 88 non-coastal Alaska south of the Brooks Range sites (provided as supplementary material)^43^. *SWE* across this wide range of sites is relatively conserved (14.49 +/− 5.4 cm) and encompasses the SWE values from at our research sites (~12 cm for both the coniferous and deciduous ecosystems)^43^. We utilized an Adirondack snow sampler to measure SWE at each of our four sites. Five snow density and 50 snow depth measurements were averaged following the combination technique of Rovansek *et al*.^44^. Measurements were conducted in mid-March, prior to the ablation period, to capture the maximum snow water equivalent.

## Additional Information

**How to cite this article**: Young-Robertson, J. M. *et al*. Deciduous trees are a large and overlooked sink for snowmelt water in the boreal forest. *Sci. Rep.* **6**, 29504; doi: 10.1038/srep29504 (2016).

## Supplementary Material

Supplementary Information

## Figures and Tables

**Table 1:** Landscape area for each ecosystem type in Alaska and the shared watersheds in western Canada, including the Yukon River watershed, fraction of snowmelt water taken up prior to leaf-out, snowmelt water uptake during the period of maximum tree water content (between snowmelt and leaf-out), and projected snowmelt water uptake with an increase in deciduous cover.

		ecosystem type	
		deciduous tree dominated	coniferous tree dominated
**landscape area (km**^**2**^**; %)**		579,568; 61.6%	360,980; 38.4%
**fraction of snowmelt uptake (%)**	**2013**	21.3% [18.9%, 24.1%]	0.65% [0.55%, 0.75%]
	**2014**	25.0% [22.4%, 28.0%]	0.62% [0.53%, 0.73%]
**snowmelt water uptake (m**^**3**^) **at maximum water content (prior to leaf-out)**	**2013**	17.79 × 10^9^[16.45 × 10^9^, 19.15 × 10^9^]	33.84 × 10^7^[29.79 × 10^7^, 38.42 × 10^7^]
	**2014**	20.88 × 10^9^[19.57 × 10^9^, 22.22 × 10^9^]	32.70 × 10^7^[28.68 × 10^7^, 37.30 × 10^7^]
**snowmelt water uptake(m**^**3**^**) with an increase in deciduous tree cover by 1, 5, 10, or 15%:**	**1%**	19.6 × 10^9^[18.3 × 10^9^, 21.0 × 10^9^]	‒
	**5%**	20.4 × 10^9^,[19.0 × 10^9^, 21.9 × 10^9^	‒
	**10%**	21.4 × 10^9^[19.9 × 10^9^, 22.9 × 10^9^]	‒
	**15%**	22.3 × 10^9^[20.8 × 10^9^, 23.9 × 10^9^	‒

**Table 2:** Results presented are scaled estimated means and 95% credible intervals for the deciduous trees for the period of maximum water content prior to leaf-out to a given number of days after leaf-out are shown.

	days	2013	days	2014
**water transpired from time of maximum water content to days following leaf-out (m**^**3**^ **water)**	**3**	2.20 × 10^9^[4.09 × 10^8^, 4.00 × 10^9^]**12.3% [2.4**–**22.0%]**	**4**	4.01 × 10^8^[0, 1.08 × 10^9^]**1.94% [0**–**5.12%]**
	**16**	3.23 × 10^9^[2.04 × 10^9^, 4.51 × 10^9^]**18.1% [11.6%, 24.3%]**	**8**	1.09 × 10^9^[4.05 × 10^8^, 1.76 × 10^9^]**5.16% [1.94%, 8.35%]**
	**33**	4.57 × 10^9^[3.09 × 10^9^, 6.14 × 10^9^]**25.7% [17.8%, 33.5%]**	**27**	1.73 × 10^9^[1.04 × 10^9^**8.35% [5.16%, 11.5%]**
	57*	5.15 × 10^9^[4.03 × 10^9^, 6.29 × 10^9^]**28.8% [23.5%, 34.1%]**	35*	1.98 × 10^9^[1.31 × 10^9^, 2.71 × 10^9^]**9.46% [6.27%, 12.6%]**

The non-bold values are the tree water volumes (m^3^) and the bold values are the percentage of the maximum tree water volume transpired. *minimum tree water content occurred 57 and 35 days after leaf out in 2013 and 2014, respectively.
