# Supplementary material for: Deciduous trees are a large and overlooked sink for snowmelt water in the boreal forest
Source: Sci Rep. 2016 Jul 12;6:29504. doi: 10.1038/srep29504 (PMC4941571; doi:10.1038/srep29504)
Supplement: Supplementary Information [file srep29504-s1.doc]

Title: Deciduous trees are a large and overlooked sink for snowmelt water in the boreal forest

Authors: J. M. Young, W. R. Bolton, U. Bhatt, J. Cristobal, and R. Thoman.

Supplementary Material: The non-coastal sites from which SWE data were utilized.

The non-coastal Alaskan sites from which SWE data (Fisher 2015) were utilized are: Alexander Lake, Archangel Road, Arctic Village, Bettles Field, Big Fish Lake, Bonanza Creek, Bonanza Forks, Borealis, Boundary, Caribou Creek, Caribou Mine, Caribou Snow Pillow, Chicken Airstrip, Chisana, Chuathbaluk, Circle City, Circle Hot Spring, Clearwater Lake, Cleary Summit, Coldfoot, olorado Creek, Disaster Creek, Edgar Creek, Fairbanks Field Office, Faith Creek, Fort Greely, Fort Yukon, Fossil, French Creek, Gerstle River, Gold King, Granite Creek, Graphite Lake, Grouch Creek, Haystack Mountain, Hess Creek, Hollikachuk, Horsefly Creek, Horsepasture Pass, Jatahmund Lake, Kantishna, Lake Minchumina, Lake Todatonten, Little Chena Bottom, Little Chena Ridge, Little Nelchina, Lost Chicken Hill, McGrath, Menotl Creek, Mentasta Pass, Middle Innoko, Mission Creek, Monahan Flat, Monsoon Lake, Monument Creek, Mt. Fairplay, Mt. Ryan, Munson Ridge, Paradise Hill, Paxson, Point Mackenzie, Ptarmigan Creek, Ptarmigan Airstrip, Purkeypile Mine, Rock Creek Bottom, Rock Creek Ridge, St. Anne Lake, Sanford River, Seven Mile, Shaw Creek Flats, Stack Pup Creek, Table Mountain, Tazlina, Telaquana Lake, Teuchet Creek, Thirty Mile, Tok Junction, Tozikaket, Tyone River, Upper Chena, Upper Chena Pillow, Upper Innoko, Upper Wood River, Wapoo Hills, Windy Gap, Wolf, Yankee Slough, and Yetna River.

**Reference cited**

Fisher, D. B. Snow course SWE averages for Alaska 1971 - 2000, <http://ambcs.org/pub/sc_sum_ak/SNOWCOURSE.HTM> (2015), (Date of access: 09/09/2015).
